# Optimizing Prescription of Chinese Herbal Medicine for Unstable Angina Based on Partially Observable Markov Decision Process

**DOI:** 10.1155/2013/532534

**Published:** 2013-09-03

**Authors:** Yan Feng, Yu Qiu, Xuezhong Zhou, Yixin Wang, Hao Xu, Baoyan Liu

**Affiliations:** ^1^Department of General Practice, Anzhen Hospital, Capital Medical University, Beijing 100029, China; ^2^Department of Cardiology, Xiyuan Hospital, China Academy of Chinese Medical Sciences, Beijing 100091, China; ^3^School of Computer and Information Technology, Beijing Jiaotong University, Beijing 100044, China; ^4^China Academy of Chinese Medical Sciences, Beijing 100700, China

## Abstract

*Objective*. Initial optimized prescription of Chinese herb medicine for unstable angina (UA). *Methods*. Based on partially observable Markov decision process model (POMDP), we choose hospitalized patients of 3 syndrome elements, such as *qi* deficiency, blood stasis, and turbid phlegm for the data mining, analysis, and objective evaluation of the diagnosis and treatment of UA at a deep level in order to optimize the prescription of Chinese herb medicine for UA. *Results*. The recommended treatment options of UA for *qi* deficiency, blood stasis, and phlegm syndrome patients were as follows: Milkvetch Root + Tangshen + Indian Bread + Largehead Atractylodes Rhizome (ADR = 0.96630); Danshen Root + Chinese Angelica + Safflower + Red Peony Root + Szechwan Lovage Rhizome Orange Fruit (ADR = 0.76); Snakegourd Fruit + Longstamen Onion Bulb + Pinellia Tuber + Dried Tangerine peel + Largehead Atractylodes Rhizome + Platycodon Root (ADR = 0.658568). *Conclusion*. This study initially optimized prescriptions for UA based on POMDP, which can be used as a reference for further development of UA prescription in Chinese herb medicine.

## 1. Introduction

Unstable angina (UA) is the clinical status between exertional stable angina and acute myocardial infarction [1]. In recent years, traditional Chinese medicine (TCM) got wealthy experience and remarkable achievements in the treatment of UA. However, there are still problems in how to scientifically evaluate the clinical efficacy of different clinical treatment recommendations and how to convert many personalized experiences into certain standardized treatment plans to follow.

Treatment regimen optimization based on partially observable Markov decision process (POMDP) is the course of using scientific methods of computation to find the most economical and convenient treatment program with best clinical efficacy among many treatment options [2–4]. The certain amount of knowledge accumulated and discovered from the massive clinical data and TCM treatment experience can not only verify the existing experience and theory of TCM that may also find some new treatment experience. In this study, we will try to use data mining methods based on large-scale, nonexternal control observational clinical data in practice to seek and find the optimized TCM treatment prescription.

## 2. Materials and Methods

### 2.1. Subjects

From September 2009 to February 2011, a total of 2212 hospitalized subjects were enrolled from China-Japan Friendship Hospital affiliated to National Health and Family Planning Commission (Beijing, China) (589 cases), Xiyuan Hospital affiliated to China Academy of Chinese Medical Science (Beijing, China) (362 cases), Guang Anmen Hospital affiliated to China Academy of Chinese Medical Science (Beijing, China) (298 cases), Wangjing Hospital affiliated to China Academy of Chinese Medical Science (Beijing, China) (97 cases), Dongzhimen Hospital affiliated to Beijing University of Chinese Medicine (Beijing, China) (193 cases), Beijing Integrative Medicine Hospital (Beijing, China) (121 cases), Beijing Chinese Medicine Hospital affiliated to Capital Medical University (Beijing, China) (186 cases), Beijing Anzhen Hospital affiliated to Capital Medical University (Beijing, China) (42 cases), Beijing Tongren Hospital affiliated to Capital Medical University (Beijing, China) (43 cases), People's Hospital affiliated to Peking University (Beijing, China) (41 cases), Huairou District Hospital of Traditional Chinese Medicine (Beijing, China) (138 cases), and Tongzhou District Hospital of Traditional Chinese Medicine (Beijing, China) (102 cases). All selected subjects fulfilled the diagnosis of unstable angina. Ethical approval was granted by the Ethics Committee of China-Japan Friendship Hospital (Beijing, China). Informed consent was obtained by each patient participating in this study.

### 2.2. Diagnostic Standard

UA diagnostic standard referred to the UA diagnosis and treatment recommendations by the China Cardiovascular Association released in 2007 [5]. According to the characteristics of angina pectoris, typical ECG changes, exercise treadmill ECG, Holter, cardiac scintigraphy, coronary angiography and risk elements to make the judgment in order to improve the accuracy of diagnosis. Diagnostic standards of TCM syndromes referred to the following references: (1) the TCM standards of coronary heart disease by China Association of Integrative Medicine Association for cardiovascular diseases [6] and (2) the chest stuffiness and pains dialectical standards in Chinese internal medicine [7].

### 2.3. Inclusion Criteria

The inclusion criteria were as follows: (1) meet the diagnostic criteria; (2) previous history of old myocardial infarction or coronary angiography confirmed at least one coronary stenosis ≥50%, (3) hospitalized patients with UA as first western medical diagnosis, (4) age, sex, druged use, and concomitant diseases are not limited, and (5) a signed informed consent.

### 2.4. Exclusion Criteria

The exclusion criteria were composed of five conditions: (1) occurred end-point events in 1-year fellow, (2) cancer and immune system diseases, (3) pregnant or lactating women, (4) serious diseases of liver, kidney, and hematopoietic system, and (5) patients with allergies or psychosis.

### 2.5. End-Point Events Criteria

(1) The primary end-point events are cardiovascular death, nonfatal myocardial infarction, revascularization (including intervention, coronary artery bypass grafting); (2) secondary end-point events are stroke, rehospitalization for ACS, heart failure, and other thrombotic complications.

### 2.6. Western Medical Treatment

According to the UA diagnosis and treatment recommendations by China Cardiovascular Association released in 2007 [5], patients were given conventional western drug therapy including anti-ischemic (nitrates, beta-blockers, calcium antagonists, and ACE inhibitors), antiplatelet (aspirin and/or clopidogrel), anticoagulation (heparin or low molecular weight heparin), and statin drugs.

### 2.7. Observed Indicators and Methods

Doing clinical information collection, verification, supplementary, pre-processing and data mining, and analysis for data acquisition process in accordance with the unified design of UA. Using TCM clinical research data platform for individualized treatment, qualified clinical researchers after training and examination took patients' hospital stay information and entered them into the database through clinical information collection system; then the professionals of the school of Computer Science in Beijing Jiaotong University convert the data for extraction, washing, and analysis. To specify the TCM syndrome following the rules of “Diagnostics of Chinese Medicine” [8] and “TCM syndrome differential diagnosis” [9], such as “phlegm stagnation syndrome,” “phlegm dampness syndrome,” “phlegm stasis syndrome” unified as “turbid phlegm syndrome,” break down the combined TCM syndromes into basic TCM syndrome elements. For example, break down “*qi* and *yin* deficiency syndrome” into syndrome elements as “*qi* deficiency syndrome” and “*yin* deficiency syndrome.” When encountering difficulty in distinguishing and differentiating TCM syndromes, to discuss and resolve it under the guidance of experts and references, telephone followups were enrolled one year after the end of the event.

In order to simplify the data and find common rules, we elected the core prescription medicine as the object of analysis, and the extraction of core Chinese herbal medicine applied the complex network mining method. To divide five main symptoms of patients with UA: “*chest tightness, chest pain, heart palpitations, shortness of breath, and fatigue*” into “*no, light, medium, and heavy*” four grades and to observe the changes of the main symptoms for patients during hospitalization every 1-2 days after admission time with 5 times for each, use cluster analysis method to unify symptoms.

### 2.8. Statistical Analysis

Using Oracle 9.0 g to converse the demographic data, clinical features, syndromes, and drug treatment data, while general material use frequency statistical analysis. Getting use of the data mining platform built in the major project of the Beijing Municipal Science and Technology Commission for the prevention and treatment of major diseases to optimize the prescription of Chinese herbal medicine and doing POMDP mining analysis by Beijing Jiaotong University researchers with data mining tools. The level of average discounted reward (ADR) will take the degree of main symptoms improvement as a standard to evaluate the clinical efficacy of different treatment options.

## 3. Results

### 3.1. The Demographic Information of the Subjects

A total of 2212 (1341 males and 871 females) cases of subjects, aged more than 40 years, were included in this study. This group of subjects included 1061 cases of patients complicated with hypertension, 514 cases of patients complicated with diabetes, 513 cases of patients complicated with hyperlipemia, 328 cases of patients complicated with old myocardial infarction, and 219 cases of patients complicated with cerebral infarctions. The demographic data is shown in Table 1.

### 3.2. TCM Syndrome Distribution in Patients with UA

Among the 2212 UA patients, the common Chinese syndrome elements were as follows: blood stasis (1931 cases, 87.3%), *qi* deficiency (1140 cases, 51.5%), turbid phlegm (1059 cases, 47.9%), *yin* deficiency (412 cases, 18.6%), *qi* stagnation (148 cases, 6.7%), *yang* deficiency (65 cases, 2.9%), heat (60 cases, 2.7%), and blood deficiency (23 cases, 1.0%).

### 3.3. Core Medicine of Chinese Herbal Prescription (Figures 1, 2, 3, and Table 2)

Due to the requirements in the number of patients for model, we study the treatment options for patients with UA in three TCM syndrome elements as “*qi* deficiency,” “blood stasis,” and “turbid phlegm.” Use complex network clustering method to screen the core Chinese herbal medicine of three TCM syndrome elements. With all the Chinese herbal medicine used in the treatment options of a syndrome element as the nodes, the medicine in compatibility has “interconnected” feature, and the number of “interconnected” is the medicine “related frequency”. The most frequently related nodes with other medicine have the most critical role in all the medication of this syndrome element. Thus, the densest nodes are the core prescription medicine.

### 3.4. Optimization and Efficacy Evaluation of Prescriptions for Different TCM Syndrome Elements in Patients with UA (Table 3)

 Applying POMDP model to calculate the ADR of different prescriptions, the largest ADR prescription of TCM syndrome element can get the maximum benefit in symptom improvement and long-term efficacy. Among the prescriptions for 3 different TCM syndrome elements, the combination of “Milkvetch Root + Tangshen + Indian Bread + Largehead Atractylodes Rhizome” is the optimizing prescription of TCM syndrome element “*qi* deficiency” for patients with UA, the combination of “Danshen Root + Chinese Angelica + Safflower + Red Peony Root + Szechwan Lovage Rhizome, Orange Fruit” is the optimizing prescription of TCM syndrome element “blood stasis” for patients with UA, and the combination of “Snakegourd Fruit + Longstamen Onion Bulb + Pinellia Tuber + Dried Tangerine peel + Largehead Atractylodes Rhizome + Platycodon Root” is the optimizing prescription of TCM syndrome element “turbid phlegm” for patients with UA. 

For commonly used names of Chinese herbal medicine, see Table 4.

## 4. Discussion

With the development of TCM, researches in optimizing prescription of Chinese herbal medicine are concerned by more and more scholars. Clinical formulation optimization is the process of using certain methods to improve clinical medicine prescriptions and make them applied in clinical practice. The commonly used methods at present are mainly two approaches as generalizing empirical formulation after optimization and applying data mining model to promote the optimized formulation [10].

The target of empirical formulation optimization is to study the experienced prescriptions promoted by some expert or clinician by modern medical research methods such as randomized control trail (RCT) in order to prove the reliability and practicality, providing evidence for related field of formulation optimization. This method is more stringent and convincing in formulation optimization design, and the optimizing prescriptions for the study are derived from the established treatment ones. However, cases collecting, observing, and other series of clinical research process need a large amount of human and material resources, which increases the cost of study; these many prescriptions with very good clinical applications are difficult to discover and research. In addition, for the more rigorous research process, the applicable conditions of the results received strict limits. In clinical practice, the efficacy in patients is affected by a variety of complex factors, which should be the results of a variety of drugs or interventions, making the extrapolation of experience prescriptions subject to certain restrictions.

Applying data mining model to optimize the prescriptions has got manifold attempts in clinical practice [11–14]; the most common method is a dynamic programming strategy. Its purpose is to seek the best solution in many prescriptions applying optimization techniques. Its data are from the clinical data of real world, and the data collection is carried out in parallel with the clinical course. As long as establishing the regulation of data entry, the entire data acquisition does not require large-scale acquisition process, and the time is mainly spent in data computing, which greatly saves the cost of the study to facilitate the continuous optimization of the prescriptions. Furthermore, this dynamic process is a combination of man and machine model; the strict mathematical operation is carried out at the same time when doing empirical evaluation. The actual process can be combined with expert consensus and evaluation to select the best treatment prescriptions given by computer for the most appropriate patient. This process can also be used for the discovery of clinical prescriptions or provide a scientific and rational arithmetic verification process for the summary of experience prescriptions, which may be very meaningful in the future.

POMDP model [15] is a dynamic decision model based on Markov process promoted by the Russian mathematician Markov after some improvements. Hauskrecht and Fraser [16] used POMDP in treatment prescription of ischemic heart disease and did a decision successfully in many cases. Maillart et al. [17] made analysis in the frequency of X-rays and treatment options for breast cancer patients from the cost-benefit viewpoint with this model. Zhang et al. [18] established POMDP model for prostate biopsy decisions. These explorations all provide practical basis for solving problems of sequence decision with POMDP model in the medical field. This study selected this research strategy to optimize prescriptions of Chinese herbal medicine for patients with UA, which is an exploration on the methodology.

 Concerning POMDP modeling requirements, this study was modeled following the dynamic clinical decision-making process to make the degree of remission in the symptoms for patients with UA and long-term end-point events as efficacy evaluation indicators for the choice of the optimal prescriptions; at the same time, it evaluated the original core prescription and extracted optimal treatment prescriptions as optimization recommendations of patients with UA in a certain TCM syndrome element for clinical reference.

Based on existing data, applying POMDP to compare the prescriptions of patients with same TCM syndrome element and no long-term end-point event, we found that optimizing prescription recommendation for “*qi* deficiency” patients is “Milkvetch Root + Si junzi decoction without Radix Glycyrrhizae,” prescription of “blood stasis” recommended “Danshen Root + Tao Hong Siwu decoction plus Orange Fruit without rehmanniae radix,” prescription of “turbid phlegm” recommended “Gualou xiebai banxia decoction plus Dried Tangerine peel, Largehead Atractylodes Rhizome, Platycodon Root”. Those are recommendations from strict mathematical model stimulating dynamic TCM prescription process, which are in line with conventional clinical thinking and put forward proposals worthy of strict clinical research.

Besides that, the prescriptions derived from real clinical data are experiences and summaries of clinical practice with considerable clinical significance. The proposals are in conformity with the clinical normal circumstances and prove the reliability and operability of optimizing prescription method in efficacy evaluation on the other hand.

It should be noted that the rigorous mathematical comparison method using in this study to observe short-term and long-term efficacy of prescriptions in order to discover preliminary optimizing recommendations is limited to the number of patients, indicators, follow-up time, and so on that the extension value should apply modern medical research methods such as case-control trail to do retrospectively summarized comparison or large-scale, multicenter, and large sample RCT for further verification to increase the level of evidence-based medicine. However, in complex data of clinical practice, the efficacy of the patients is affected by many complicated factors; this formulation optimization idea still has great significance in efficacy comparison and screening treatment plan, and it also provides us an optimizing prescription method for clinical practice-based data worthy of further study.

## Figures and Tables

**Figure 1:**
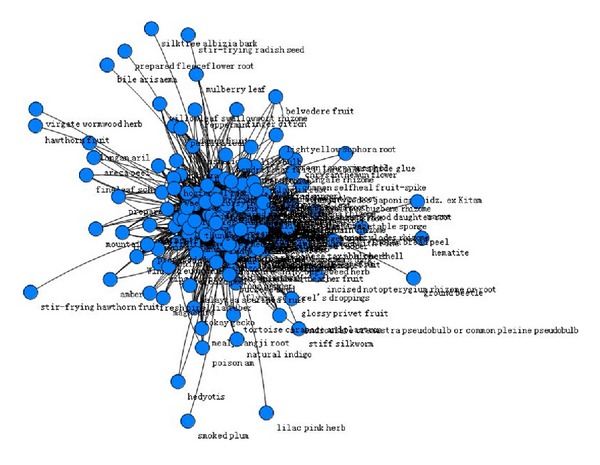
Overall situation of Chinese herbal prescription medicine for TCM syndrome element “*qi* Deficiency.”

**Figure 2:**
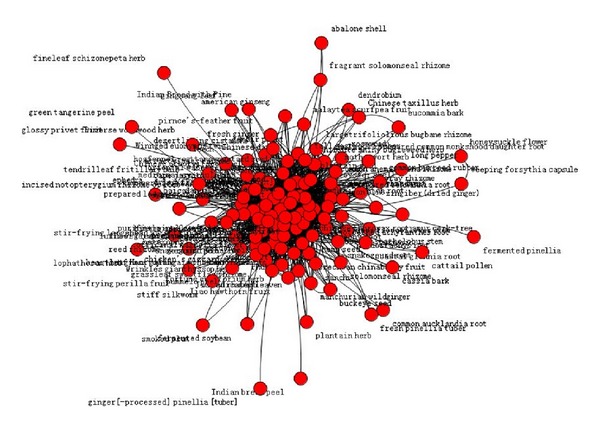
Overall situation of Chinese herbal prescription medicine for TCM syndrome element “blood stasis.”

**Figure 3:**
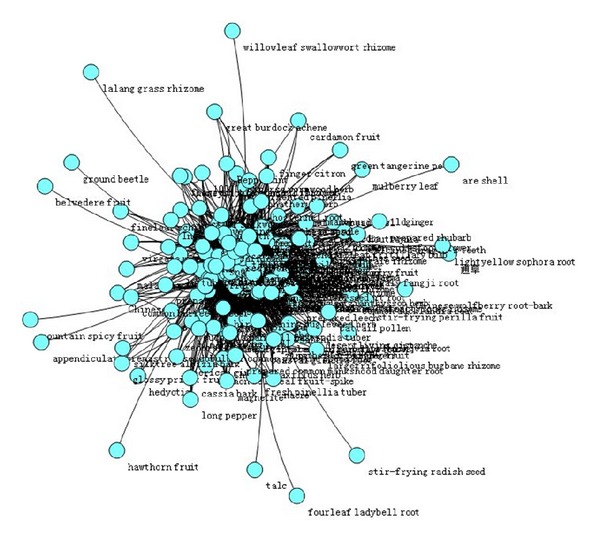
Overall situation of Chinese herbal prescription medicine for TCM syndrome element “turbid phlegm.”

**Table 1:** The demographic information of the subjects.

Items	Demographic information
Gender	
Males (case (%))	1341 (60.6%)
Females (case (%))	871 (39.4%)
Age (years)	61.2 ± 13.6
Hypertension (case (%))	1061 (48.0%)
Diabetes	514 (23.2%)
Hyperlipemia	513 (23.1%)
Old myocardial infarction	328 (14.8%)
Cerebral infarctions	219 (9.9%)

**Table 2:** Core medicine of Chinese herbal prescription.

Types of TCM syndrome element	Core medicine of Chinese herbal prescription
*Qi* deficiency	Tangshen, Heterophylly Falsestarwort Root, Largehead Atractylodes Rhizome, Milkvetch Root, Radix Glycyrrhizae, Indian Bread, and Chinese Date

Turbid phlegm	Pinellia Tuber, Snakegourd Fruit, Indian Bread, Dried Tangerine peel, Longstamen Onion Bulb, Largehead Atractylodes Rhizome, Platycodon Root, Golden Thread, Bamboo Shavings, Wrinkled Giant Hyssop + Herba Eupatorii, Wrinkled Giant Hyssop, Orange Fruit + Pinellia Tuber

Blood stasis	Szechwan Lovage Rhizome, Yanhusuo, Danshen Root, Peach Seed, Safflower, Chinese Angelica, Turmeric Root Tuber, Red Peony Root, Turmeric Root Tuber + Red Peony Root, Peach Seed + Radix bupleuri, Peach Seed + Platycodon Root, Szechwan Lovage Rhizome + Orange Fruit

**Table 3:** Optimizing prescription of different TCM syndrome elements for UA patients.

Types of TCM syndrome slement	Optimizing prescription	ADR
*Qi* deficiency	Milkvetch Root + Tangshen + Indian Bread + Largehead Atractylodes Rhizome	0.96630

Blood stasis	Danshen Root + Chinese Angelica + Safflower + Red Peony Root + Szechwan Lovage Rhizome Orange Fruit	0.76

Turbid phlegm	Snakegourd Fruit + Longstamen Onion Bulb + Pinellia Tuber + Dried Tangerine peel + Largehead Atractylodes Rhizome + Platycodon Root	0.658568

**Table 4:** Commonly used names of Chinese herbal medicine.

Chinese name	English name	Latin name
Dangshen	Tangshen	Radix Codonopsis
Huangqi	Milkvetch Root	Radix Astragali
Fuling	Indian Bread	Poria
Baizhu	Largehead Atractylodes Rhizome	Rhizoma Atractylodis Macrocephalae
Danshen	Danshen Root	Radix Salviae Miltiorrhiae
Danggui	Chinese Angelica	Radix Angelicae Sinensis
Honghua	Safflower	Flos Carthami
Chishao	Red Peony Root	Radix Paeoniae Rubra
Chuanxiong	Szechwan Lovage Rhizome	Rhizoma Chuanxiong
Zhiqiao	Orange Fruit	Fructus Aurantii
Gualou	Snakegourd Fruit	Fructrs Trichosanthis
Xiebai	Longstamen Onion Bulb	Bulbus Allii Macrostemi
Banxia	Pinellia Tuber	Rhizoma Pinelliae
Chenpi	Dried Tangerine peel	Pericarpium Citri Reticulatae
Jiegeng	Platycodon Root	Radix Platycodi
Taizishen	Heterophylly Falsestarwort Root	Radix Pseudostellariae
Gancao	Radix Glycyrrhizae	Radix Glycytthizae
Dazao	Chinese Date	Fructrs Jujubae
Huanglian	Golden Thread	Rhizoma Coptidis
Zhuru	Bamboo Shavings	Caulis Bambusae in Taeniam
Huoxiang	Wrinkled Giant Hyssop	Agastache rugosa
Peilan	Herba Eupatorii	Eupatorium fortunei
Yanhusuo	Yanhusuo	Rhizoma
Taoren	Peach Seed	Semen Persicae
Yujin	Turmeric Root Tuber	Radix Curcumae
Baishao	White peony Alba	Raidix Paeoniae Alba
Chaihu	Radix bupleuri	Bupleurum chinense
Shengdi	Rehmanniae radix	Rehmannia glutinosa Libosch
